# Factors that hinder community participation in developing and implementing comprehensive council health plans in Manyoni District, Tanzania

**DOI:** 10.3402/gha.v8.26461

**Published:** 2015-06-01

**Authors:** Emmanuel G. Kilewo, Gasto Frumence

**Affiliations:** 1 Regional Health Management Team (RHMT), Singida Region, Tanzania; 2 Department of Development Studies, School of Public Health and Social Sciences, Muhimbili University of Health and Allied Sciences, Dar es Salaam, Tanzania

**Keywords:** community participation, health planning, Tanzania

## Abstract

**Background:**

Decentralization of public health planning is proposed to facilitate public participation in health issues. Health Sector Reform in Tanzania places emphasis on the participation of lower level health facilities and community in health planning process. Despite availability of policies, guidelines, and community representative organs, actual implementation of decentralization strategies is poorly achieved. This study intended to find out factors that hinder community participation in developing and implementing Comprehensive Council Health Plan (CCHP).

**Materials and methods:**

A qualitative approach was conducted in this study with key informants from Health Facility Governing Committees (HFGC), Council Health Service Board (CHSB), and Council Health Management Team (CHMT). Data were collected using in-depth interviews. Data generated were analyzed for themes and patterns.

**Results:**

Factors that hindered community participation included lack of awareness on the CCHP among HFGC members, poor communication and information sharing between CHMT and HFGC, unstipulated roles and responsibilities of HFGC, lack of management capacity among HFGC members, and lack of financial resources for implementing HFGC activities.

**Conclusions:**

The identified challenges call for policy makers to revisit the decentralization by devolution policy by ensuring that local governance structures have adequate resources as well as autonomy to participate in planning and managing CCHP in general and health facility plans in particular.

Community participation is regarded as an important tool for successful health sector development and it has been talked about since the mid-1950s (1, 2). It is advocated for providing a mechanism for potential beneficiaries of health services to participate in the design, implementation, and evaluation of activities, with the aim of increasing responsiveness, sustainability, and efficiency of health services (3, 4).

In 1978, the Alma-Ata Declaration set principles to guide the planning, implementation, and evaluation of community-oriented health programs. One of the principles states that ‘People have the rights and duty to participate individually and collectively in the planning and implementation of their health care’ (5, p. 3).

Until the 1970s, the process of planning development activities in many countries including developing countries was centrally controlled. Having failed to achieve the expected development from the centralized planning system, policy makers and planners adopted a decentralized planning and implementation approach (6). Thus, when many developing countries started reforming their health sectors, decentralization became an important component of the reforms.

In Tanzania, the health sector reforms began in 1994 with the goal of improving access, quality, and efficiency of service delivery. This involved establishment of Council Health Service Boards (CHSBs), Health Facilities Governing Committees (HFGCs), and the introduction of a new formula of fiscal allocation to councils to enhance, among other things, good governance and community ownership in the public health care system at local levels (7, 8). Therefore, community members are expected to play an important role in developing local health plans through the established facility boards and committees. However, largely, such plans are prepared at the council level by Council Health Management Team (CHMT). In practice, the bottom-up planning has been difficult because communities do not have the opportunity or required capacity (8, 9).

Although the Government of Tanzania has made great efforts to reform the health care system by developing comprehensive policies and guidelines, there are still challenges in terms of accountability, community voice, information reporting, and feedback. Many governing bodies do not exist in some councils, and where they exist, they are just symbolic as they do not fully fulfill their roles and responsibilities (8, 9). Furthermore, little is known about the attitudes and characteristics of the local leaders, particularly members of the CHSBs and HFGCs, who form part of the health planning and decision-making process. This situation complicates further efforts to design effective interventions (10). The process of developing Comprehensive Council Health Plan (CCHP) shows that the HFGCs are consulted in the initial stage of health planning, which involves identification of priorities and needs to be included in the annual plans. The CHSBs and CHMTs are responsible organs for receiving and reviewing the annual plans and budget projections from the health facilities (hospital, health centers, and dispensaries). The CHSBs and CHMTs are also responsible for incorporating these plans into CCHP before submitting them to the Full Council and the Regional Secretariat for further review and approval (11). The HFGCs have a role of facilitating the Health facility Management Teams (HFMs) in planning and managing health initiatives in areas under their jurisdiction (12).

Despite the various efforts aiming at ensuring that communities participate in deciding about their affairs including health issues, operationalization of such efforts is poorly done. Thus, this study aimed at exploring factors that hinder Community Participation in Developing and Implementing CCHP.

## Methodology

### Study design

**A qualitative approach** was employed in this study to allow in-depth interviews and discussion with key informants about the factors influencing community participation in developing CCHP. The Manyoni district was considered a case study for exploring views and ideas from the members of facility governing committees, CHSBs, and CHMTs who have real-life experiences (13) in participating in health planning at the community level.

### Study area

The study was conducted in Singida Region, Manyoni District Council. This district was selected because it is one of the districts in Singida region which, according to the regional CCHP Assessment Report (14), has low involvement of lower level health facilities in planning processes of developing CCHP compared to other districts in the Region.

Manyoni district covers an area of 28,620 Km^2^. It borders Mbeya and Iringa Region to the south, Tabora Region to the west; Ikungi District to the north, and Bahi District in Dodoma Region to the east. Administratively it has five divisions, 30 wards, 96 villages,^1^ 356 hamlets, and 66,618 households. The district has a total population of 296,763 people (15).

There are 53 lower level health facilities (health centers and dispensaries). Of these, 42 are public dispensaries, three are public health centers and eight are dispensaries owned by faith-based organizations (FBOs). Given the limited resources and time, two wards were purposively selected for this study. One public health center was selected from each ward and two dispensaries were selected from the first ward whereas three public dispensaries were selected from the second ward.

### Data collection techniques

This study employed a qualitative method of data collection which involved conducting in-depth interviews with key informants. An interview guide with questions focused on achieving the study objectives was developed to guide the interviews. The researchers selected this type of study design based on the type of information desired. A qualitative research uses a naturalist approach, which tries to understand phenomena in context-specific settings; it gives details and insights of participants’ experiences of the world (16). In-depth interviews with key informants were found to be suitable for the study because it sought to explore the views and experiences of those who are directly dealing with planning and implementation of health facilities and council health plans. The interviews were carried out in May 2013 and each interview lasted between 60 and 90 min and was held in the offices of the respondents or special rooms provided by the district medical officer (DMO) or in-charge of a health facility. A total of 18 key informants were interviewed for this study.

A thematic guide was developed to guide interviews. The guide comprised of questions on the awareness of community participation in health planning, roles and responsibilities of CHSB, HFGCs and CHMT, information sharing among governance structures, management capacity, and availability of resources. The interviews were performed by two interviewers in Kiswahili to reduce language barriers.

### Data analysis

The study employed a thematic approach in analyzing data. Data were coded without essentially fitting them into a pre-existing researchers’ analytic pre-conception. We used an inductive approach to identify themes as they emerge from the data (17). The data were analyzed manually through reading and re-reading the transcripts until they had a clear understanding of the content. Reviewing the transcripts was done simultaneously with coding the data by listing down phrases that captured emerging concepts. These concepts were further analyzed based on the research objectives to categorize their similarities and differences as well as identifying the main emerging themes.

### Ethical issues

Muhimbili University of Health and Allied Sciences (MUHAS) Research and Publication Committee granted ethical clearance to conduct the study. Formal permission in the district was obtained from District Executive Director and DMOs, Wards Executive Officer, and Villages Executive Officer. Written consent was obtained from participants after explaining to them about study objectives, methodology, and benefits. Respondents were also assured of confidentiality of all information disclosed to the researchers.

## Findings

The analysis of the factors influencing community participation in the development and implementation of CCHP has generated five main categories, which explain why it has been difficult for participatory organs at the community level to participate fully in the planning and implementation process of health activities in these areas. These categories include: lack of awareness on CCHP among members of HFGCs, poor means of communication and information sharing between CHMT and HFGCs on CCHP, little knowledge among HFGCs on their roles and responsibilities, limited capacity among HFGCs due to lack of training, and lack of financial resources to support the implementation of HFGC activities.

### Awareness on community participation

The majority of participants had heard about community participation and they associated it with empowerment or involvement of the community in health activities or programs as narrated by some respondents from governing committees of both dispensaries and health centers:

… Aaah! What I know is that community participation is the process of involving people in deciding about their own affairs. (KI 11: member of dispensary GC)

### Awareness on CCHP

The majority of the respondents reported that they had never heard of CCHP. Some participants from HFGC also did not know about the existence of such a plan. A respondent from the health center governing committee said:

What I can say is that I do not know about CCHP and I see this is new object to me and to my committee; maybe there is another language which is used to describe it, but since my appointment to this committee I have never been informed or taught anything about CCHP. (KI 2: chairperson of health center governing committee)

Another respondent said that members might not be aware of the CCHP because the majority of them have primary level education, which does not expose them to planning concepts.

Our findings have revealed that almost 70% of members of HFGCs had primary level education. Only in-charges of health facilities who were also secretaries of HFGC had secondary level of education.

It was highly surprising to find that even the members of the CHSBs, who are responsible for endorsement of the council plan, were not aware of CCHP. This situation can be substantiated by the following comment:

I have not read or heard it as this is my first time, what I can say is that the CHSB has not yet participated in endorsing or implementing CCHP. (KI 3: member of CHSB)

### The influence of training on awareness on CCHP among HFGCs members

The training of CCHP emerged as an important theme which may enhance or lower awareness of CCHP among CHMTs and HFGCs members. For instance, in-charges of health facilities, who are also secretaries of HFGC, were partially informed on CCHP and plans of health facilities and most of them had heard about the CCHP through attending certain workshops/training. Despite such awareness, they were unable to explain or clarify it well. A member of a health center governing committee had this to say:

I heard about CCHP when I was attending a workshop at the regional headquarters but I do not know in details about it. (KI 1: in-charge of health center)

Awareness of CCHP was found to be higher among CHMT members due to the fact that they are the ones responsible for preparing and coordinating the implementation of the CCHPs. CHMT members further declared that there was low awareness on CCHP and health facility plans among HFGCs members because such committee members have not been trained on health planning processes. One CHMT member said:

CCHP is an annual activity plan for health sector developed by involving various stakeholders who also participate in its implementation. HFGCs are not aware of CCHP, as we have not given them any training on the subject, however, we are planning to train them. (KI 2: CHMT member)

Lack of training has also been reported to have an effect on the performance of HFGC members. In this regard, the HFGC members claimed that after their appointment as members of the committees they never received any capacity building training concerning roles, responsibilities, and management in general. For that reason, they lack financial and management skills to perform their duties in the development and implementation of CCHP as narrated by one of the respondents:

We were elected to form HFGC in 2010. Nobody received any kind of training related to our responsibilities as committee members, and we just work from experience. I think this is the high time for us to get training on management and planning topics. (KI 12: chairperson of dispensary GC)

### Involvement in the development and implementation of CCHP

All interviewed HFGC members reported that for many years, the lower level health facilities (health centers and dispensaries) are operating without budgets and they were not conversant with the annual activities and budgets, which have been developed, approved, and implemented by CHMT on their behalf. In addition, participants from HFGC claimed that they never received any feedback from DMOs concerning the proposed budget and plans submitted to the council through in-charges of health facilities.

Since I became a member of HFGC I never saw or heard that our committee is consulted or involved in any stage of preparing what you called CCHP. Just to remind you, the facilities do not have a budget or bank account, so how can they be involved? (KI 11: member of dispensary GC)

Contrary to the information obtained from the members of HFGC, CHMT members reported that they usually involve staff from lower level health facilities during the preparation of CCHP.

What I know is that the CHMT usually involves the lower health facilities staff in the CCHP preparation but the problem is that the HFGC members are not aware of what is going on due to the fact that they have never been oriented or trained on health planning. (KI 2: CHMT member)

Another member of CHMT provided an additional explanation as to why there was low involvement of HFGC members in the CCHP development process:

The real situation is that involvement of HFGC in developing the CCHP is still low due to the fact that the health facility plan and CCHP are required to be developed in English; therefore if you look at the composition of the HFGC members, with exception of in-charges of health facility, other members have Primary Level of Education. (KI 1: CHMT member)

As regards the implementation of the planned activities in the CCHP, the study found that the lower level health facilities staff are not aware of the annual facility budget, district budget, annual planned activities and even the action plans for implementing various activities in the CCHP. A respondent from the lower level health facility reported that they had no budget or any activities assigned from district level for them to implement at the facility level.

… In short, we do not implement any activities outlined in the CCHP. (KI 15: in-charge of dispensary)

Our respondents said that HFGC were rarely involved in the implementation of some activities such as construction/rehabilitation of facility buildings. Such involvement occurred because the implementation of the projects had a requirement that facility committees must endorse the implementation of such activities as explained by one of the HFGC chairperson:

Yeah to tell the truth, we cannot say that we are implementing CCHP activities but in 2008 we were involved only in rehabilitation of our dispensary. (KI 6: chairperson of dispensary GC)

From the CHMT, it was found that the only CCHP activity implemented directly at the health facility level is the ordering and receiving of drugs through an Integrated Logistic System (ILS).

Through ILS the CHMT is not responsible for ordering medicines for the health facilities, the facilities implement this activity on their own. (KI 2: CHMT member)

### Communication and information sharing

The key informants from HFGC pointed out that the higher-level authority does not disseminate important information for developing and implementing facility plans and CCHP to health centers and dispensary committees. This situation has resulted into low awareness of the development process of CCHP by the HFGC members. A respondent from the dispensary governing committee said:

… the existing system of information sharing between CHMT and HFGCs is not adequate. For example, last year (2012) we held a HFGC meeting and proposed for rehabilitation of our dispensary, thereafter we forwarded our minutes to the DMOs but up to now, he has not responded. (KI 15: in-charge of dispensary)

These findings reveal that the only existing formal communication system between CHMT and HFGC is when the CHMT give instructions to lower level health facilities through letters. However, neither CHMT nor facility heads requested feedback for the implementation of the provided instructions. Other formal means of communication stipulated in the guidelines are never practiced. One member of the CHMT stated:

At the beginning of the planning period we usually write letters to all facility in-charges asking them to provide their priorities, surprisingly, some do respond and some do not. (KI 1: CHMT member)

Respondents cited supportive supervision as the major means of information sharing between the district level and facility level. However, the supportive supervision checklist used by CHMT to supervise health facilities does not provide room for discussing issues relating to CCHP. In addition, during the supportive supervision CHMT do not provide feedback or hold special meetings with the HFGC for discussing various issues related to the facilities including the health plans. A key respondent from a dispensary governing committee expressed this concern:

We hear from our in-charge that DMO and his team are regularly coming for supportive supervision but we just wonder why they do not want to involve the whole committee in discussion and feedback. This is the only chance for us to discuss issues related to health plan and expenditure of Community Health Fund and user fees. (KI 6: chairperson of dispensary GC)

### Roles and responsibilities

The findings revealed that almost all HFGC members had not been oriented or trained on their roles and responsibilities. However, the committees are reportedly performing or involved partially with other roles such as sensitizing the community to join the CHF, receiving and opening new drug kits, creating awareness on health problems, monitoring disease outbreaks, and giving advice to communities on health matters. During the interviews, it was observed that most of the participants from HFGC except the secretary of the committee were not aware of the roles and responsibilities of developing and implementing CCHP and health facility plans. When asked to explain their roles and responsibilities in the development of health plans, one respondent said:

For my knowledge I know we are supposed to participate in development and implementation of health plans but we have not performed this role because we have not yet been trained on issues related to health plans. (KI 8: in-charge of dispensary)

CHMT respondents also confirmed the findings that members of the HFGC were not trained or oriented with their duties and responsibilities:

Honestly, we have not trained or oriented any of the HFGC members in our district on their roles and responsibilities. Actually it is a challenge and we are working on our budgets to tackle it. (KI 2: CHMT member)

The responses from district level were almost similar to those provided by HFGC members, that the guidelines on roles and responsibilities of HFGCs have not been disseminated though they have been distributed to some of the health facilities. A member from CHMT had the following to say:

We have not disseminated the guidelines to any health facility. However, the village authorities were provided with instructions on how to formulate the committees. (KI 1: CHMT member)

### Management capacity of HFGC

Members of HFGC reported that after their appointment as members of the committees, they never received any capacity building training concerning management in general and planning in particular. For that reason they lack management skills and knowledge to perform their duties in development and implementation of CCHP as narrated by one of the respondents:

We were elected to form HFGC in 2010. Nobody received any kind of training related to our responsibilities as committee members, and we just work from experience. I think this is the high time for us to get training on management and planning topics. (KI 12: chairperson of dispensary GC)

CHMT respondents also confirmed the findings that members of the HFGC were not trained or oriented in their duties and responsibilities particularly including those related to management of health facilities and their involvement in health planning:

Members of the CHMT reiterated that lack of funds for conducting capacity building programs including training on management and planning has largely contributed to lack of management capacity, especially planning skills, among HFGC members.

During the interviews respondents also indicated that there was no uniformity in the process of appointing community members into HFGC and the process appears to vary from one health facility to another. One respondent had the following to say:

The doctor in charge informed me that I was one of the members of the HFGC but as far as I could remember, I never applied or contested for this post. (KI 3: member of health center GC)

On the contrary, another respondent reported:

I sent my application on the post to the village government and during the general village meeting I was elected and become one of the members. (KI 13: chairperson of health center GC)

The interview indicated that there is little support from government authorities at the district and village level to support the HFGCs in implementation of their roles especially in preparing and implementing the health facility plans. This might have been contributed by a negative attitude of CHMT toward HFGCs in the sense that once they become competent with their functions, CHMT will control all resources and power. One of the respondents said:

Maybe the CHMT fears that once we are capable to undertake our functions they will lose control of the resources which are supposed to be managed by ourselves. This is because currently they are planning and managing everything for us. (KI 1: in-charge of health center)

### Financial resources

Respondents reported lack of a specific budget in the Council Health Plan for financing HFGC activities. This study found that lack of financial resources to support the implementation of HFGC activities in the CCHP not only hinders the participation of the HFGC in developing and implementing CCHP but also hinders the effective functioning of these committees, including lack of timetables for conducting meetings as required in the guidelines.

Yes it's true we are not budgeting for any HFGC activities due to shortage of funds but we really know this situation has negative impact to the performance of HFGC. (KI 1: CHMT member)

Some participants showed concern that lack of financial resources for paying allowances to HFGC members when they perform their duties has led them to become dormant or ineffective in participating in the implementation of various activities. They underscored that allowances act as catalysts and motivators for members of HFGC to work hard. Lack of budget negatively affects the implementation of HFGC activities as narrated by one of the respondents:

Lack of transport allowance for attending meeting has significantly affected the functioning of our committee as people tend to escape HFGC scheduled regular meetings for this reason. (KI 13: chairperson of health center GC)

Another respondent claimed that:

Some members were interested to be members of HFGC with the intention of receiving allowances and other payments, therefore the absence of such allowances has discouraged them to participate in HFGC activities. (KI 2: CHMT member)

Furthermore, HFGC members mentioned that despite the fact that they were not aware as to how often the committee should meet, they lack budget to organize meetings to discuss challenges facing health facilities and find solutions. This situation has contributed to poor performance of HFGCs as narrated by one respondent:

You know lack of budget for supporting the organization of the HFGCs meetings; it is not possible to have effective committees as originally intended. (KI 10: in-charge of health center)

Some respondents stated that HFGC have played little or no role in monitoring collection and utilization of user fees and CHF in their facilities but they are aware that health facilities are responsible for collecting funds through user fees and CHF.

Can you imagine HFGC is not responsible for managing financial resources at the facility level? We have been collecting user fees and CHF and submitting all to the DMOs office. No member of HFGC knows how these resources are being utilized. (KI 8: in-charge of dispensary)

The comments from CHMT on collection of user fees and CHF were not different from those of HFGC members, as reported by of the CHMT members:

Yes the facilities submit to us the collection of user fees and CHF because lower level facilities have not yet started to operationalize bank account; they are supposed to write the request of expenditure of their money after getting the permission of HFGC. However, some do and other facilities do not apply for money. I do not know what is the cause of such failure? I am not sure it is due to lack of knowledge or something else. (KI 2: CHMT member)

## Discussion

This section is structured into the main themes that emerged from the findings of this study which include: low awareness of HFGC on participation in health planning, poor communication and information sharing between CHMT and HFGC, lack of awareness of the roles and responsibilities of HFGC, lack of management capacity, and lack of financial resources allocated to support implementation of HFGC activities.

### Low awareness of HFGCs on participation in health Planning

The study revealed that most of the HFGC members were unaware of the concept of CCHPs and how they were supposed to participate in developing and implementing CCHP at the health facility level. They reported of never hearing or having been introduced to CCHP and its processes before. This is consistent with the findings from other studies (10, 18) which reported that lack of awareness about community participation contributes to low participation of community members in developing and implementing various health projects. This study found that low levels of education among HFGC members contributed to low awareness on the theme of participation of HFGCs in developing and implementing health plans. Given the low level of education for most of the committee members, it was difficult for them to analyze issues and fully participate in planning of health activities. Other studies (19–23) reported that a high level of education among key actors in health systems increased confidence and influenced participation in decision making in health activities and intervention.

### Poor communication and information sharing between CHMT and HFGC

The findings indicated that there was poor communication and information sharing between CHMT and lower level health facilities in all subjects related to the CCHP. The HFGCs members have partial or no information related to the development and implementation of facility plans and CCHP, which was mainly caused by lack of joint management or planning meetings between the two organs. The observed poor communication and information sharing has resulted in poor involvement of HFGC in development and implementation of CCHP. Evidence from a randomized field experiment on community-based monitoring in Uganda has shown that community members were unable to participate fully in decision making including monitoring of service providers and types of services provided because there was poor communication from the health facility to the community with regards to services provided to the community. The evidence show further that after the intervention of improving the sharing of information between trained health staff and community members, there was improvement of community members in making decisions about their health needs as well as monitoring the way service providers deliver services (24). A study done in Canada reported similar findings, which emphasize that for community members to raise the voices in decision making processes, some important factors must be in place including sharing of experiences between health service recipients or health boards and the service providers (25).

Moreover, this study found that the HFGCs did not conduct their scheduled quarterly meetings as per the CHSB's establishment tool of 2001. HFGC members mentioned two main reasons for failure to conduct the meetings, namely lack of budget to finance the meetings and lack of timetable and knowledge on how often they are supposed to meet. These factors contribute to poor information sharing among committee members on all issues pertaining to health facilities including those related to developing and implementing health facility plans and CCHP. These findings are consistent with other findings (8, 26), which revealed that lack of meeting allowances has a significant effect on committee functioning.

### Lack of awareness of the roles and responsibilities of HFGC leads to poor participation in the development of CCHP

This study revealed that the majority of the HFGC members were not aware of their roles and responsibilities. It was further observed that almost all HFGC members were not oriented on their roles and responsibilities in managing health services delivery. The existence of HFGC whose members do not have a clear understanding of their roles and responsibilities contributed to the weak participation of the lower level health facilities in development and implementation of CCHP.

The study found that there were no official documents available at the lower level health facility level regarding roles and responsibilities, which could guide the daily operations of the committees. This situation led to establishment of structures with no real mandate. These results correspond to findings from other studies (21, 26, 27), which found that uncertainty about roles and responsibilities resulted in ineffectiveness in HFGCs’ performance. Similarly other studies (28, 29) pointed out that the confusion about roles and responsibilities hinder community participation in health projects. The United Republic of Tanzania (URT) (30) stipulates clearly that the granted power and degree of autonomy as well as clear definition of roles of the health boards were important factors in their success.

### HFGC members lack management capacity

Lack of management capacity particularly in planning skills among HFGC members was a common concern raised in this study. Similar findings were reported in other studies (9, 31–36), which noted that lack of capacity building programs contribute to inactiveness of different workers in performing their roles and responsibilities. They further reported that a classical issue in decentralization is lack of capacity characterized by insufficient human resources, inadequate training, and poor management as well as insufficient management system and procedure. In this study, we have found that most of the members of HFGC had primary education level, which seems to be insufficient to make them competent in performing their roles without exposing them to appropriate capacity building programs through training. Furthermore, lack of funds for conducting training programs, lack of uniformity in the procedure of appointing members of HFGC, and negative attitude of CHMT toward strengthening the HFGC to perform their duties have also contributed to lack of management capacity especially planning skills among HFGC members.

### Lack of financial resources allocated to support implementation of HFGC activities

The study found that there were no funds allocated for running committee activities. The members of HFGC were volunteering in performing their duties. This contributed to the failure of the committee to function as per government guidelines. It was learnt that lack of autonomy at the lower level health facilities in controlling budget and their annual plans has partly contributed to the failure of HFGC to manage properly the collection of user fees and CHF. Furthermore, lack of funds allocated for organizing planning sessions between facility management and HFGC members largely contributed to poor performance of HFGC activities. These results are similar to those found in other studies (37–39) who pointed out that the capacity of such committees to perform their functions was constrained by inadequate resources.

## Conclusions

This study concludes that HFGCs are potentially instrumental organs to participate in the development of facility plans and CCHP. The Government of Tanzania established HFGCs as part of its efforts to implement a bottom-up planning approach in the development and implementation of CCHPs. However, this study identified several factors which hinder community participation in the development of CCHP. These include: lack of awareness among HFGC members; lack of awareness on the roles and responsibilities of HFGC; poor means of communication and information sharing between CHMT and HFGC, lack of management capacity of members of HFGC, and lack of financial resources for HFGC activities in their respective areas. There is a need for the national and district health authorities to address these problems so as to provide an enabling environment that will ensure better involvement of community and lower level health facilities in the development and implementation of various health plans for better health outputs. Among other things, the national and local authorities could design awareness intervention campaigns on community participation and health planning, making clear definitions of functions, roles, and responsibilities of HFGC; design and implement a capacity development program for HFGCs for the purposes of raising (HFGC) community's knowledge on CCHP particularly focused on community participation in development and implementation of health plans through HFGC; and establish strong communication between HFGCs and CHMT on matters related to CCHP. Other important measures include improving supportive supervision from the CHMT to facility level, ensuring proper dissemination of official documents related to HFGC and CCHP, allocation of financial resources to facilitate HFGC activities, and ensuring that newly elected HFGCs’ members are well prepared through orientation programs for members to understand their roles and responsibilities.

The identified challenges facing the HFGCs in the development and implementation of CCHP calls for policy makers both at national and district levels to revisit the decentralization by devolution policy by ensuring that local governance structures have adequate resources as well as autonomy to participate in planning and managing CCHP in general and health facility plans in particular.

## Footnotes

1 A division is the largest subdivision of a district and it is subdivided into a number of wards. A ward is a largest subdivision of a division and it is subdivided into streets in the urban areas and into villages in the rural areas. A village is a largest subdivision of a ward. Public health centers are located at the ward level while public dispensaries are situated at the village level.
